# Fatal Human Alphaherpesvirus 1 Infection in Free-Ranging Black-Tufted Marmosets in Anthropized Environments, Brazil, 2012–2019

**DOI:** 10.3201/eid2804.212334

**Published:** 2022-04

**Authors:** Tais M. Wilson, Jana M. Ritter, Roosecelis B. Martines, Hannah A. Bullock, Pamela Fair, Kay W. Radford, Isabel L. Macêdo, Davi E.R. Sousa, Alexandra A.B. Gonçalves, Alessandro P. Romano, Pedro H.O. Passsos, Daniel G. Ramos, Gabriela R.T. Costa, Karina R.L.J. Cavalcante, Cristiano B. de Melo, Sherif R. Zaki, Marcio B. Castro

**Affiliations:** Centers for Disease Control and Prevention, Atlanta, Georgia, USA (T.M. Wilson, J.M. Ritter, R.B. Martines, P. Fair, K.W. Radford, S.R. Zaki);; University of Brasília, Brasília, Brazil (T.M. Wilson, I.L. Macêdo, D.E.R. Sousa, A.A.B. Gonçalves, C.B. de Melo, M.B. Castro); Synergy America Inc., Atlanta (H.A. Bullock);; Brazilian Ministry of Health, Brasília (A.P. Romano, P.H.O. Passos, D.G. Ramos, K.R.L.J. Cavalcante);; Environmental Health Surveillance Directorate of the Federal District, Brasilia (G.R.T. Costa)

**Keywords:** human alphaherpesvirus 1, One Health, zoonoses, fatal, pathology, histopathology, immunohistochemistry, transmission electron microscopy, PCR, marmoset, anthropized environments, viruses, Brazil

## Abstract

Human alphaherpesvirus 1 (HuAHV1) causes fatal neurologic infections in captive New World primates. To determine risks for interspecies transmission, we examined data for 13 free-ranging, black-tufted marmosets (*Callithrix penicillata*) that died of HuAHV1 infection and had been in close contact with humans in anthropized areas in Brazil during 2012–2019. We evaluated pathologic changes in the marmosets, localized virus and antigen, and assessed epidemiologic features. The main clinical findings were neurologic signs, necrotizing meningoencephalitis, and ulcerative glossitis; 1 animal had necrotizing hepatitis. Transmission electron microscopy revealed intranuclear herpetic inclusions, and immunostaining revealed HuAHV1 and herpesvirus particles in neurons, glial cells, tongue mucosal epithelium, and hepatocytes. PCR confirmed HuAHV1 infection. These findings illustrate how disruption of the One Health equilibrium in anthropized environments poses risks for interspecies virus transmission with potential spillover not only from animals to humans but also from humans to free-ranging nonhuman primates or other animals.

The coronavirus disease (COVID-19) pandemic has brought the One Health concept to the forefront of global health. From an infectious disease standpoint, the focus of One Health is often on how human activity expanding and encroaching on wildlife habitats may adversely affect humans through spillover of pathogens from wildlife reservoirs. However, the opposite—transmission of pathogens from humans to wildlife—is also possible in these situations. In Brazil and other countries, destruction and alterations of natural habitats and deforestation driven by human activities such as agricultural and urban expansion force some nonhuman primate (NHP) populations to live in anthropized areas, intensifying interactions between humans and NHP species and increasing the risk for interspecies transmission of agents of infectious diseases (*1*). 

The black-tufted marmoset (*Callithrix penicillata*) is one example of an NHP now well-adapted to human-altered environments. Marmosets are naturally found in the Brazilian Savanna and Caatinga Biome and are commonly commensal in urban and periurban areas (*2*); close human–marmoset interactions (i.e., feeding) are common. Because these settings are suitable for interspecies transmission of pathogens, infectious disease surveillance of NHPs provides an invaluable opportunity to detect emerging and reemerging zoonotic and anthroponotic diseases as well as predict pathogen spillover events.

Alphaherpesviruses usually cause asymptomatic or mild infections in their natural hosts but are often associated with severe illness after cross-transmission to new species (*3*). Human alphaherpesviruses (HuAHVs) consist of 2 closely related viruses, HuAHV types 1 and 2. Transmission of both HuAHV types generally requires intimate contact between actively infected and susceptible persons. Humans are natural hosts for HuAHV1, also known as herpes simplex virus 1, an alphaherpesvirus that is endemic in human populations (*4*). Natural fatal HuAHV1 infections are well-documented for New World primates that are in close contact with humans as pets and in captive conditions such as zoos and biomedical research centers (*5*–*15*). In contrast, HuAHV1 infections in free-ranging New World primates in anthropogenic environments are rarely reported and are limited to isolated outbreaks in urban areas (*16*) and in a state conservation park (*17*).

Captive New World primates are highly susceptible to HuAHV1 infection, and fatal disease frequently develops, characterized by mucocutaneous, facial, and oral erosions/ulcerations and meningoencephalitis (*5*–*15*). However, reports of HuAHV1 infection in free-living animals in anthropized environments are limited. We conducted a retrospective study to determine pathologic, immunohistochemical, and ultrastructural features of infection; molecular identification of the virus; and epidemiologic aspects of fatal outbreaks and isolated cases of HuAHV1 virus infection in free-ranging black-tufted marmosets in anthropized areas of the Federal District and surrounding areas of Brazil.

## Methods

### Case Selection

To select cases of suspected fatal herpetic infection, we reviewed 1,042 NHP necropsies performed during 2012–2019 and archived by the Veterinary Pathology Laboratory at the University of Brasilia, Federal District, and the Regional Reference Laboratory of the Brazilian Ministry of Health that performs necropsies to diagnose yellow fever as part of the National Surveillance Program of Yellow Fever Epizootics in NHP of the Brazilian Ministry of Health. For all cases, we retrieved epidemiologic information, clinical data, pathologic findings, and image data from submission forms, necropsy reports, archived images, and formalin-fixed paraffin-embedded (FFPE) tissue blocks.

### Histopathologic Evaluation

Necropsy tissue samples from 13 NHPs with clinically suspected emerging infectious diseases were fixed in 10% formalin, embedded in paraffin, sectioned, and stained with hematoxylin and eosin. We evaluated the following samples to identify histopathologic changes consistent with suspected HuAHV infection: brain (meningitis, perivascular inflammation, neuropil inflammation with or without prominent neutrophilic component, neuronal necrosis, neuronophagy, reactive gliosis, glial nodules, and inclusion bodies within neurons and glial cells); tongue (epithelial ballooning degeneration, acantholysis, ulceration, necrosis, subepithelial inflammation, intranuclear inclusion bodies within the mucosal epithelium and syncytial cells); and liver (hepatocellular coagulative necrosis, hepatocellular eosinophilic intranuclear viral inclusion bodies, and multinucleated giant cells [syncytia]). We also recorded histopathologic changes in other organs.

### Immunohistochemistry and Transmission Electron Microscopy

We performed immunohistochemistry (IHC) for HuAHV types 1 and 2 on FFPE tissues. We used a polymer-based colorimetric indirect immunoalkaline phosphatase method in deparaffinized tissue sections after rinsing the tissue sections in 1X Tris‐buffered saline with Tween 20 (1X TBS‐T; Thermo Fisher Scientific, https://www.thermofisher.com). Tissue sections were digested with 0.1 mg/mL Proteinase K (Roche, https://www.sigmaaldrich.com) in 0.6 M Tris/0.1% CaCl_2_ for 15 min and blocked in Background Punisher (Biocare Medical, https://biocare.net) for 10 min. Slides were incubated with a rabbit polyclonal anti-HuAHV1 and 2 antibody biological products maintained at CDC at 1:2,000 dilution, and attached antibodies were detected with Mach 4 Universal AP Polymer Kit (Biocare Medical) and Permanent Red Chromogen (Cell Marque/Millipore Sigma, https://www.cellmarque.com). We counterstained slides with Mayer hematoxylin (Polyscientific, https://www.polyrnd.com) and placed coverslips by using an aqueous mounting medium (Polysciences, Inc., https://www.polysciences.com). Appropriate positive and negative controls were run in parallel.

We also processed FFPE sections from the brain, liver, and tongue of 3 NHPs with extensive immunohistochemical evidence of HuAHV for transmission electron microscopy (TEM) analysis by using an on-slide technique. In brief, 4-μm sections of FFPE tissue affixed to glass slides that correlated with areas positively labeled for HuAHV by IHC were deparaffinized in xylene, then rehydrated and fixed in 2.5% buffered glutaraldehyde. Samples were postfixed with 1% osmium tetroxide, stained en bloc with uranyl 132 acetate, dehydrated, and embedded in Epon-Araldite resin with dibutyl phthalate (Electron Microscopy Sciences, https://www.emsdiasum.com). Epoxy resin–embedded sections on the glass slide were immersed in boiling water and removed from the slides with a razor blade; areas of interest were glued onto electron microscopy blocks. We stained ultrathin sections with uranyl acetate and lead citrate and examined them on either a Thermo Fisher/FEI Tecnai Spirit or Tecnai BioTwin electron microscope (*18*).

### Real-Time PCRs for HuAHV1 and HuAHV2

We conducted real-time PCR for HuAHV 1 and HuAHV2 on FFPE samples with confirmed herpesvirus immunohistochemistry results. We extracted DNA from 16-μm paraffin-embedded brain or liver tissue sections from each animal by using a QIAamp UCP Pathogen Mini Kit (QIAGEN, https://www.qiagen.com) according to the manufacturer’s recommendations. Fluorescence resonance energy transfer (FRET) technology was conducted to discern HuAHV1 from HuAHV2 infections in a real-time PCR targeting the glycoprotein B, UL27 gene. The 2-probed system discriminates HuAHV1 and HuAHV2 according to analysis of the melt curves (*19*). We considered a sample to be HuAHV1 positive if the melt temperature was 56°C and HuAHV2 positive if 63°C. We included cases positive by FRET PCR for HuAHV1 or HuAHV2 in our study.

To amplify a 147-bp fragment of the glycoprotein B, UL27 gene from the genome of either HuAHV1 or HuAHV2, w used HuAHV1 and HuAHV2 primers (HuAHV-1/2 forward primer, 5′-TTG AAG CGG TCG GCG GCG TA- 3′; HuAHV-1/2 reverse primer, 5′-GTC CAC CTC CTC GAC GAT GC- 3′) along with the detection probe (5′-LC Red 640- GCG ACT GGC GAC TTT G- 3′-phos-cpg) and the anchor probe (5′-GGT AGC CGT AAA ACG GGG ACA TGT A- 3′-fam-cpg). PCR was performed in a 20-μL reaction volume with 0.5 μmol/L of the 50 μmol/L primer stocks, 0.2 μmol/L of the detection probe, 0.1 μmol/L of the anchor probe, and HotStarTaq DNA Polymerase and 1X QuantiTect Probe PCR Master Mix (both from QIAGEN). The PCR had the following cycling conditions: a hot start (95°C), touchdown (10 cycles of denaturation at 95°C, annealing at 62°C, and an extension at 72°C), amplification (40 cycles of denaturation at 95°C, annealing at 52°C, and an extension at 72°C), cooling at 40°C, and 1 melt cycle at 95°C. We considered a sample to be as considered positive if the melt temperature was ≈56°C for HuAHV1 and 63°C for HuAHV2. We included cases positive by FRET PCR for HuAHV1 or HuAHV2 in our study.

## Results

### Real-Time PCR, Epidemiologic, and Clinical Findings

**Figure 1:**
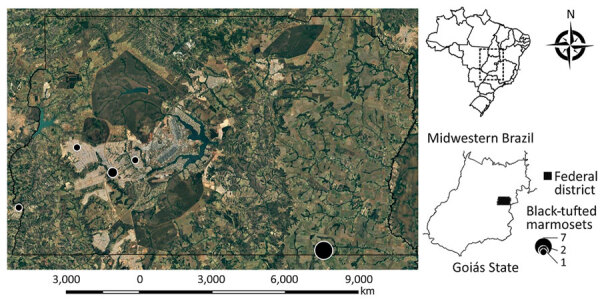
Locations of urbanized free-ranging black-tufted marmosets with fatal human alphaherpesvirus 1 infections, Federal District, Goiás, Brazil, 2012–2019. Circles indicate known locations of outbreak 1 (7 animals), outbreak 2 (2 animals), and 3 isolated cases. Insets indicate location of Federal District (black shading) in Goiás state and Goiás state in Brazil. Figure adapted from Google Maps (https://www.google.com.br/maps).

Of the 1,042 retrieved NHP cases, HuAHV fatal infection was morphologically diagnosed for 18 black-tufted marmosets (Table 1). Of these, 5 (27.8%) were from captive conditions and excluded from our study, and for the remaining 13 free-ranging marmosets, HuAHV1 was detected in all tissues tested by real-time PCR. HuAHV2 was not detected in any sample. Marmoset deaths were distributed in urban (38.5%) and peri-urban areas (61.5%) of the Federal District and Goiás State, Brazil (Figure 1). Available information indicated close contact with humans for 7 marmosets. Investigations of the probable infection site frequently showed close contact between marmosets and humans, including local residents feeding fruit to animals.

**Table 1:** Epidemiologic, clinical, and gross necropsy features for free-ranging black-tufted marmosets that died of human alphaherpesvirus 1 infection, Brazil, 2012–2019

Feature	No. affected/total (%)*
Sex	
M	10/13 (77)
F	3/13 (23)
Age group	
Juvenile	6/13 (46)
Adult	7/13 (54)
Epidemiologic features	
Peri-urban location	7/12 (58)
Urban location	5/12 (42)
Outbreak	9/13 (69)
Isolated case	4/13 (31)
Known contact with humans	7/13 (54)
Clinical signs	
Neurologic changes	
Muscular tremors	4/12 (33)
Depression	3/12 (25)
Recumbency	2/12 (17)
Seizures	2/12 (17)
Anisocoria	1/12 (8)
Ataxia	1/12 (8)
Nystagmus	1/12 (8)
Not specified (witnessed)	5/12 (42)
Oral cavity	
Salivation	8/12 (66)
Bleeding	1/12 (8)
Gross findings	
Lymphadenomegaly	4/13 (58)
Nonulcerative glossitis	5/13 (38)
Ulcerative glossitis	4/13 (31)
Facial erythema and rash	1/13 (7)

*Denominators indicate numbers of animals for which information was available.

Two separate HuAHV1 outbreaks during 2012–2019 involved 9 (69%) of the 13 animals, (7 animals in outbreak 1; 2 animals in outbreak 2); the other 4 (31%) had isolated cases. In outbreak 1, an entire family group of marmosets became ill and died. Information about clinical signs was available for 12 animals. Neurologic signs were most frequently reported and included ataxia (8%), seizures (17%), muscle tremors (33%), nystagmus (8%), and anisocoria (8%). Nonspecific clinical signs were also reported, such as depression (25%), recumbency (17%), salivation (66%), and oral bleeding (8%). Fatal cases affected adults (54%) and juveniles (46%).

### Pathology, Immunohistochemistry, and Electron Microscopy Findings

**Figure 2:**
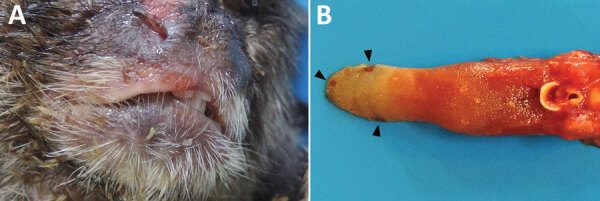
Macroscopic finding in the tongue and lip from a black-tufted marmoset with fatal human alphaherpesvirus 1 infection, Brazil, 2012–2019. A) Erosions and ulcerations on the lip. B) Glossitis with multifocal ulcers and erosions (arrowheads).

Gross changes retrieved from necropsy reports and photographic documentation revealed multiple cutaneous crusting erythematous erosions and ulcerations in the periocular region, lip, and tongue (Figure 2). Mandibular and cervical lymph nodes were often markedly enlarged. The most commonly affected organs, of those available for evaluation, were the brain (92%), tongue (69%), and liver (8%) (Table 2). Histopathologic findings in other organs included reactive lymph node hyperplasia (77%) and necrosis of splenic white pulp germinal centers (15%). IHC demonstrated HuAHV1 antigen in the brain of 12 (92%) marmosets, the tongue of 9 (69%), and the liver of only 1 (8%). HuAHV1 immunostaining was not detected in other representative tissue samples available, including spleen (n = 13 animals), lymph node (n = 7), heart (n = 8), kidney (n = 8), lung (n = 7), esophagus (n = 1), gastrointestinal tract (n = 7), trachea (n = 1), adrenal gland (n = 1), pancreas (n = 1), and testicle (n = 4).

**Table 2:** Histopathologic features found during necropsy of free-ranging black-tufted marmosets naturallly infected with human alphaherpesvirus 1, Brazil, 2012–2019

Organ, finding	No. affected/total (%)*
Brain	12/13 (92)
Inclusion bodies within neurons and glial cells	12/12 (100)
Neuronal necrosis	12/12 (100)
Mononuclear perivascular cuffs	12/12 (100)
Neuronophagy	11/12 (92)
Reactive gliosis	11/12 (92)
Neuropil inflammation	
Mononuclear cells	10/12 (83)
Neutrophils	3/12 (25)
Intravascular leukocytosis	9/12 (75)
Nonsuppurative meningitis	9/12 (75)
Glial nodules	5/12 (42)
Reactive neurovascular endothelium	5/12 (42)
Tongue	9/13 (69)
Acantholysis	8/9 (89)
Epithelial ballooning degeneration	8/9 (89)
Epithelial intranuclear inclusion bodies	8/9 (89)
Subepithelial inflammation	8/9 (89)
Epithelial necrosis	5/9 (55)
Ulcer	4/9 (44)
Syncytial cells	2/9 (22)
Liver	1/13 (8)
Hepatocellular coagulative necrosis	1/1 (100)
Intranuclear viral inclusion bodies	1/1 (100)
Multinucleated giant cells	1/1 (100)

*Denominators indicate numbers of animals for which information was available.

#### Brain

**Figure 3:**
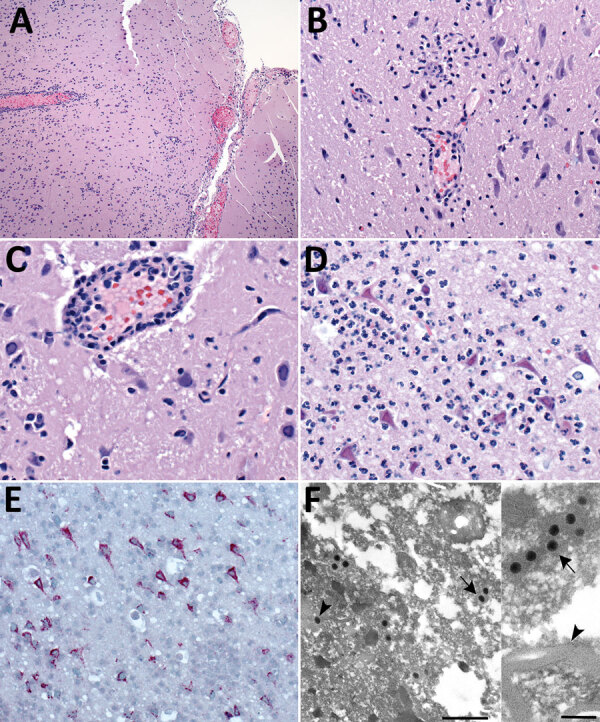
Pathologic changes in brain of free-ranging black-tufted marmosets with fatal human alphaherpesvirus 1 infection, Brazil, 2012–2019. A) Necrotizing meningoencephalitis. Hemotoxylin and eosin (H&E) stain; original magnification ×10. B) Neuronal degeneration and glial nodule. H&E stain; original magnification ×40. C) Neuronal necrosis with microglial proliferation and expansion of Virchow–Robbin spaces by lymphocytes, histiocytes, and few plasma cells. Neurons and glial cells show intranuclear inclusion bodies and prominent margination of the nuclear chromatin. H&E stain; original magnification ×63. D) Prominent neutrophilic inflammation accompanies neuronal necrosis and intranuclear inclusion bodies. H&E stain; original magnification ×63. E) Human alphaherpesvirus 1 immunostaining within neurons (immunohistochemistry; original magnification ×40). F) Intranuclear (arrowhead) and cytoplasmic (arrow) herpesvirus particles in gray matter. Transmission electron microscopy; scale bar indicate 500 nm. Inset: cytoplasmic herpesvirus particles (arrow) white matter, myelinated axon (arrowhead); scale bar indicates 200 nm.

The most frequent neuropathologic finding in the HuAHV1-infected marmosets was necrotizing meningoencephalitis (Figure 3, panel A). Findings included reactive gliosis, glial nodules, neuronal necrosis, neuronophagia (Figure 3, panel B), characteristic herpetic intranuclear eosinophilic inclusion bodies within neurons and glial cells, and prominent perivascular inflammation of the gray and white matter (Figure 3, panel C). Inflammatory infiltrates varied from moderate to severe and consisted of lymphocytes, histiocytes, neutrophils, and a few plasma cells. For 3 animals, marked neutrophilic inflammation in the neuropil was detected (Figure 3, panel D). Swollen reactive endothelial cells and intravascular leukocytosis were also seen in affected areas of neuroparenchyma. Infected neurons and glial cells showed strong cytoplasmic immunostaining for HuAHV1 (Figure 3, panel E). TEM analysis of the brain revealed viral particles morphologically consistent with herpesvirus in the white and gray matter; intranuclear and cytoplasmic viral particles were also observed (Figure 3, panels F and G).

#### Tongue

**Figure 4:**
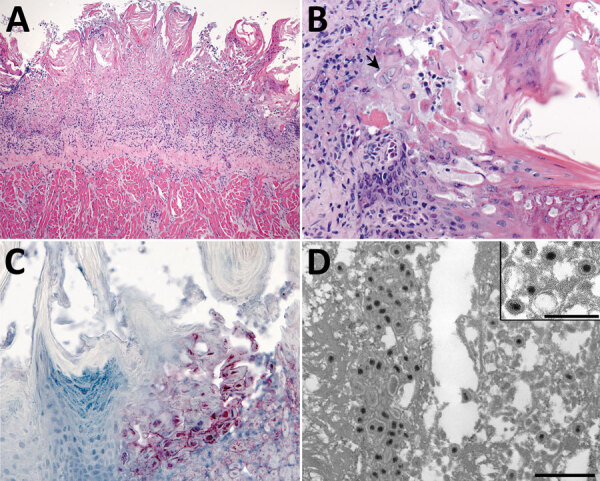
Tongue pathology in free-ranging black-tufted marmosets with fatal human alphaherpesvirus 1 infection, Brazil, 2012–2019. A) Severe necrosis of epithelium. Hemotoxylin and eosin (H&E) stain; original magnification ×10. B) Intranuclear inclusion bodies in epithelial cells at the margin of the lesion and multinucleated syncytial cell (arrow). H&E stain; original magnification ×40. C) Human alphaherpesvirus 1 immunostaining within epithelial cells in the area of necrotizing glossitis. Immunihistochemistry; original magnification ×40. D) Epithelial cell containing accumulations of herpesvirus within the cytoplasm. Transmission electron microscopy; scale bar indicates 600 nm. Inset: higher magnification image of herpesvirus particles with well-defined tegument layer in the cytoplasm; scale bar indicates 400 nm.

Histopathologic lesions in the tongue were multifocal, showing moderate to severe epithelial ballooning degeneration and acantholysis; multifocal to coalescing mucosal ulceration; necrosis with fibrin deposition (Figure 4, panel A); and subepithelial neutrophilic infiltrates with histiocytes, lymphocytes, and plasma cells. Epithelial cells showed rare multinucleation (syncytia formation) with nuclear molding and herpetic intranuclear eosinophilic inclusion bodies (Figure 4, panel B). Necrotic and surrounding ulcerated areas showed epithelial cells with marked cytoplasmic and nuclear viral immunostaining (Figure 4, panel C). Correlating with this viral immunostaining, TEM identified abundant herpesvirus in the nuclei and cytoplasm of epithelial cells (Figure 4, panel D).

#### Liver

**Figure 5:**
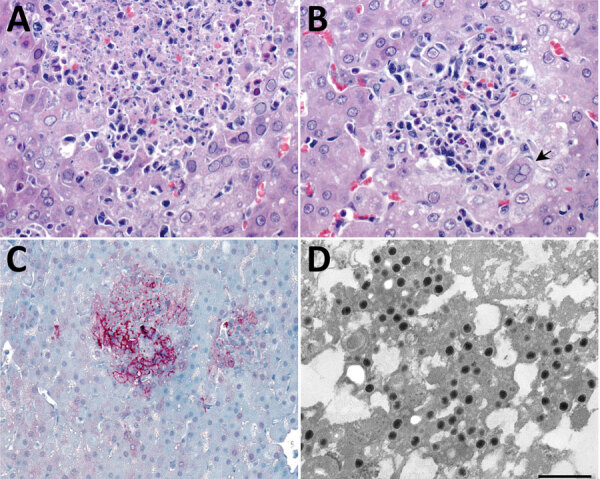
Liver pathology in free-ranging black-tufted marmosets with fatal human alphaherpesvirus 1 infection, Brazil, 2012–2019. A) Intranuclear inclusion bodies in hepatocytes at the margin of a necrotic focus. Hemotoxylin and eosin (H&E) stain; original magnification ×63. B) Multinucleated syncytial cell (arrow). H&E; original magnification ×63. C) Human alphaherpesvirus 1 immunostaining within hepatocytes in an area of necrotizing hepatitis. Immunihistochemistry; original magnification ×40. D) Herpesvirus in the cytoplasm of a hepatocyte. Transmission electron microscopy; scale bar indicates 400 nm.

Histopathologic lesions in the liver were found in only 1 of the 13 marmosets. In this animal, necrotizing hepatitis was characterized by multifocal random coagulative necrosis, few neutrophils, and scattered hemorrhage (Figure 5, panel A). Hepatocellular eosinophilic intranuclear viral inclusion bodies and multinucleated giant cells (syncytia) were also observed (Figure 5, panels B). Necrotic foci showed strong immunolabeling for HuAHV1 antigens (Figure 5, panel C). Herpesvirus was found by TEM primarily in the cytoplasm of hepatocytes; a few nuclei also showed the presence of the virus (Figure 5, panels D).

## Discussion

The expansion of human activity into natural environments can disrupt the One Health equilibrium by increasing opportunities for infectious disease transmission between humans and animals. The effect of this equilibrium on human public health is often emphasized; however, disease transmission can also threaten wildlife and biodiversity in periurban settings. The 13 synanthropic free-ranging marmosets with acute, fatal HuAHV1 infections described here were from across a densely populated, anthropized environment in Brazil. Our findings corroborate those of previous reports of HuAHV1 in captive and pet marmosets and other NHPs, from clinical and pathologic standpoints, expanding the body of knowledge of HuAHV1 infection in free-ranging marmosets and highlighting the value of strategic NHP infectious disease surveillance systems.

The natural reservoir for HuAHV1 is humans, and infection of these marmosets resulted from documented or probable close interactions with humans in urban or periurban environments. HuAHV1 infection occurs by viral host epithelial invasion through direct or indirect contact with mucocutaneous lesions or with bodily secretions/excretions from asymptomatic carriers (*3*,*6*,*8*,*20*). In at least some of these marmosets, confirmed contact involved humans directly sharing food with marmosets. In the absence of direct sharing, food scraps and garbage are potential sources of HuAHV1 infection for free-ranging marmosets and other animals in anthropized environments such as the Federal District and surrounding areas. The high number of sick and dead marmosets in 1 family of marmosets in our series and in monkey families reported elsewhere suggests that animal-to-animal transmission of HuAHV1 probably also occurs (*5*,*7*–*10*,*13*–*17*). We found numerous viral particles within oral epithelial lesions of marmosets, suggesting high mucosal viral shedding and potential for transmission to other animals (*21*). Experimental HuAHV1 infection of rhesus macaques also demonstrated ocular, nasopharyngeal, oral, fecal, and urine virus shedding (*22*).

The clinical disease observed in these animals (neurologic signs, hypersalivation, and oral bleeding) correlated with the gross findings of glossitis and facial erythema with regional lymphadenomegaly and with histopathologic features characteristic of herpes viral meningoencephalitis and stomatitis. Similar clinical and gross findings have been reported for marmosets with fatal and nonfatal HuAHV1 infection, and necrotizing meningoencephalitis and ulcerative glossitis are the histopathologic hallmarks observed in most documented HuAHV1 outbreaks among captive and free-living NHPs (*6*–*9*,*11*–*17*,*23*–*25*).

According to histologic findings, death of these marmosets is largely attributable to the neurologic effects of infection (*12*,*13*). HuAHV1 antigen detection by IHC and viral particle detection by TEM within brain lesions confirmed viral neuroinvasiveness in these animals. Animal models have shown that primary HuAHV1 infection originates in the skin or oral mucosa, usually followed by a latent stage in sensory neurons, and finally reaching the brain through the trigeminal nerve or olfactory bulb, causing lethal encephalitis (*26*). Intracellular HuAHV1 replication triggers direct cytopathic effects such as programmed cell death and necrosis (*27*). In the 13 marmosets, brain pathology also indicated that a severe inflammatory response to viral infection played a role in disease pathogenesis (*28*–*30*). In some HuAHV1 outbreaks among marmosets, however, mortality rates may be elevated even in the absence of neuropathologic changes because marmosets are highly susceptible to infection (*6*). Necrotizing hepatitis with syncytia and viral inclusions, as seen in the juvenile marmoset with liver tissue available, has only rarely been reported for HuAHV1-infected NHPs (*7*). Similarly, a severe, disseminated disease with mucocutaneous lesions, hepatitis, and encephalitis occurs only sporadically in human neonates and severely immunocompromised patients (*4*) as a consequence of high viral load in hosts incapable of limiting replication at mucosal surfaces (*31*).

As with HuAHV1, other alphaherpesviruses typically cause mild, self-limiting disease in their natural host species but severe, generalized, and often fatal disease when cross-species transmission occurs (Table 3) (*3*). Among NHPs, infections of Old World monkeys compared with New World monkeys are generally more self-limiting and fatal cases more rare (*23*–*25*,*28*,*32*,*33*). The mechanisms underlying these variations in susceptibility are not fully known but may be associated with species differences in innate immune system function or cellular DNA repair proteins needed for the virus life cycle (*34*,*35*). Cercopithecine herpesvirus 1 (also called B virus) is the most concerning in terms of zoonotic risk, resulting in fatal human infection after transmission from macaques. Human exposure is typically reported in biomedical research settings but could also occur through interactions with macaques in anthropized natural environments (*3*). Analogous to B virus cross-species infection in humans, our findings reinforce the high susceptibility and severe outcomes of cross-species HuAHV1 infections in marmosets (*6*).

**Table 3:** Primate alphaherpesviruses and interspecies disease manifestations*

Alphaherpesvirus ICTV name (common name)	Natural host species	Species with severe generalized disease	Species with self-limiting disease
Human alphaherpesvirus 1, 2 (herpes simplex virus)	Human	New World primates	Humans, Old World monkeys
Chimpanzee α-1 herpesvirus†	Chimpanzee	Unknown	Chimpanzees
Macacine herpesvirus 1 (B virus)	Macaques	Humans, African green monkeys	Macaques
Papiine herpesvirus 2 (herpesvirus papio 2)	Baboon	Unknown	Baboons
Cercopithecine alphaherpesvirus 2 (simian agent 8)	Vervet, baboon, African green monkey	Unknown	Baboon
Langur herpesvirus†	Langur	Unknown	Langur
Saimirine herpesvirus 1	Squirrel monkey	Owl monkeys, marmosets, tamarins	Squirrel monkey
Ateline alphaherpesvirus 1	Spider monkey	Unknown	Spider monkey
Human herpesvirus 3 (varicella zoster virus)	Human	Unknown	Humans, great apes
Cercopithecine alphaherpesvirus 9 (simian varicella virus)	Macaque	African cercopithecines	African cercopithecines

*ICTV, International Committee on Taxonomy of Viruses. †Not classified by ICTV (https://talk.ictvonline.org).

Viral latency with recrudescence is typical of alphaherpesvirus infections in the natural host, and latency can be associated with asymptomatic virus shedding (*20*). HuAHV1 is endemic to the human population in Brazil, and seroprevalence studies indicate high rates of infection (*36*). Serologic testing has also shown the potential for persistent infection in marmosets (*9*), implying a potential risk for spillback to humans. HuAHV1 transmission from humans to NHPs and vice versa may therefore be underrecognized; further studies are needed to determine the extent of interspecies transmission in urban and periurban settings.

In Brazil, other enzootic causes of outbreaks and acute deaths among marmosets (e.g., toxoplasmosis, rabies, and yellow fever) are included in the clinical and pathologic differential diagnosis for HuAHV infection (*37*–*39*). These other diseases are zoonotic, some of high public health concern, with possible NHP reservoir hosts. This observation exemplifies the value of public outreach and robust One Health–focused epidemiologic and pathologic surveillance programs, such as the National Surveillance Program of Yellow Fever Epizootics in NHP of the Brazilian Ministry of Health, for detecting zoonotic and anthroponotic diseases in free-ranging NHPs (*40*). Detection, monitoring, and predicting spillover events afforded by these programs enables development of rapid containment measures to prevent devastating new public health outbreaks and threats to wildlife populations.
